# Unraveling Halogen Role in Two-Step Solution Growth of Organic–Inorganic Hybrid Mixed-Halide Perovskites: Guidelines of Fabricating Single-Phase Perovskites with Predictable Stoichiometry

**DOI:** 10.1021/acsomega.4c02650

**Published:** 2024-06-05

**Authors:** Ya-Rong Lee, Yun-Ting Chung, Tsung-Yu Chiang, Ta−Li Hsieh, Yi-Hang Su, Juen-Kai Wang

**Affiliations:** † Institute of Atomic and Molecular Sciences, Academia Sinica, Taipei 10617, Taiwan; ‡ Department of Physics, National Taiwan University, Taipei 106, Taiwan; § Center for Condensed Matter Sciences, National Taiwan University, Taipei 106, Taiwan

## Abstract

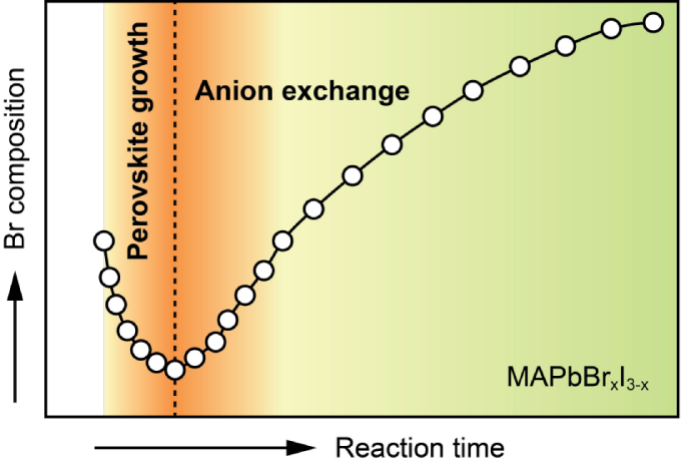

The challenge faced in optoelectronic applications of halide perovskites is their degradation. Minimizing material imperfections is critical to averting cascade degradation processes. Identifying causes of such imperfections is, however, hindered by mystified growth processes and is particularly urgent for mixed-halide perovskites because of inhomogeneity in growth and phase segregation under stresses. To unravel two-step solution growth of MAPbBr*_x_*I_3–*x*_, we monitored the evolution of Br composition and found that the construction of perovskite lattice is contributed by iodine from PbI_2_ substrate and Br from MABr solution with a 1:1 ratio rather than a 2:1 ratio originally thought. Kinetic analysis based on a derived three-stage model extracted activation energies of perovskite construction and anion exchange. This model is applicable to the growth of PbI_2_ reacting with a mixed solution of MABr and MAI. Two guidelines of fabricating single-phase MAPbBr*_x_*I_3–*x*_ with predictable stoichiometry thus developed help strategizing protocols to reproducibly fabricate mixed-halide perovskite films tailored to specific optoelectronic applications.

## Introduction

Metal-halide perovskite (MHP) materials—bearing a ABX_3_ chemical formula, where A is a univalent organic or inorganic cation, B is a bivalent metal cation, and X is a halide anion—have attracted intensive attention in the field of solar photovoltaics due to their ease of fabrication, defect tolerance,^1^ and versatile optoelectronic properties and recently have further extended their applications to light-emitting diodes, lasers, and photodetectors from infrared to X-ray.^2,3^ To date, the power conversion efficiency of perovskite solar cells (PSCs) has been rapidly improving from just 3.8% in 2009^4^ to 26.1% for single junction cells and 33.9% for tandem solar cells with crystalline silicon recently.^5,6^ Despite the dramatic progress, the obstacles on the way to commercialization are intrinsic imperfections in grown materials (defect, disorder, grain boundary, etc.) and susceptibility to external stresses (moisture, heat, radiation, and electric field),^7,8^ resulting in short device lifetime.^8−11^ Moreover, the material imperfections and external stresses are entangled and effectively enforce each other. Many recent researches have been engaged to develop new material growth procedures^12,13^ to minimize the intrinsic material imperfections so that the cascaded degradation processes would not start at the outset, but they mostly rely on speculations and trial-and-error approaches because of limited knowledge in actual happenings during material growth.^14^

The current understanding of MHP growth posits that precursor distribution and unknown intermediates involved hold the greatest responsibility for producing the aforementioned material imperfections that lead to device degradation. Such effects are particularly common in the perovskite films grown with one-step solution growth method—nucleation with premixed precursors dissolved in solvent—which has been greatly adopted in the fabrication of the active layer of PSCs.^12,15^ As a consequence, great efforts have been made to improve the film quality by optimizing the fabrication parameters,^16−20^ including selecting solvent,^21,22^ adjusting precursor concentration,^23−25^ introducing additive,^15,26,27^ tweaking loading time,^28^ and varying growth temperature.^24,29^ Despite its simplicity, high-quality films grown with the one-step method cannot be easily achieved by optimizing these fabrication parameters independently, because of complicated intermediates and processes expectantly involved (e.g., iodoplumbate complexes [PbI_n_]^m–^,^30−33^ solvent-PbI_2_-MAI intermediate phase^34−36^). In contrast, Liang et al. developed a two-step method^37^ to grow ABX_3_ perovskites with AX in solution interacting with a prepared porous BX_2_ template. Reproducible film quality and device performance have been achieved because the nucleation of the perovskite is expected to proceed within the inorganic template that can be independently optimized.

The two-step method has been reported that it is preferred for its superior reproducibility compared to the one-step method,^38−40^ requiring fewer reaction variables and is particularly advantageous for growing mixed-cation and/or mixed-anion perovskites by addressing solubility differences among precursor ions. Various mechanisms have been proposed for the two-step method,^41−46^ including transformation via MAI ion incorporation and dissolution followed by recrystallization at different concentration ranges of MAI.^25,44^ Additionally, it has been suggested to begin with a topotactic nucleation, probably preceded by MAI intercalation, and followed by dissolution of PbI_2_ structure.^45^ Recently, a three-stage mechanism involving A^+^ and X^–^ ions uptake into PbX_2_, formation of AX-PbX_2_, and subsequent conversion to APbX_3_ was introduced.^46^ However, verifying these models is challenging due to difficulties in detecting the intermediates of lacking identifiable signatures.^34^ It is commonly assumed that collaboration between halogens from the BX_2_ template and AX in solution forms an AX–BX_2_ intermediate. Yet, questions arise regarding the coexistence of A^+^ and X^–^ ions with different diffusivities and intermediate formation with a lower X^–^ supply.

A recent simulation study^47^ proposed that I^–^ from MAI undertakes a nucleophilic attack to the PbI_2_ structure to form iodoplumbates, (PbI_2_)^2-n^, where *n* = 3, 4, ..., as the primary stage of the two-step growth process. This raised the question of how such intermediates are produced if the supply of I^–^ from MAI is restrained. To decipher the respective roles of halides from MAI and the PbI_2_ framework in the two-step growth process, we exploited different halogens in AX and BX_2_. Using X-ray diffraction (XRD) and UV–vis spectrophotometry, we analyzed the bromine composition in grown MAPbBr*_x_*I_3–*x*_ perovskite and the residual PbI_2_ quantity. In addition, kinetic analysis was conducted at different reaction temperatures and a three-stage model was proposed for kinetic analysis. The study extended the model to understand the growth with mixed solution of MABr and MAI (abbreviated as MABr:MAI) at varying fractions of Br^–^, *f*_Br_s. This research yielded high-quality MAPbBr*_x_*I_3–*x*_ perovskites (*x* = 0.1 to 2.9) and established two fabrication guidelines of such mixed-halide perovskites.

In this report, we first describe the experimental methods adopted, including precursor preparation, reaction design, and characterizations of X-ray diffraction and spectrophotometry. A three-stage model is presented to explain the evolution of the extracted Br composition of MAPbBr*_x_*I_3–*x*_ perovskite films grown with the reaction scenario of PbI_2_ reacting with MABr solution. The kinetic analysis results of this model at different reaction temperatures are used to extract the activation energies of perovskite formation and halide exchange. We then describe the application of this model to the reaction scenario of PbI_2_ reacting with solution of MABr:MAI. The fabrication guidelines of mixed-halide perovskites resulting from the two reaction scenarios are provided. The issues of this study and their possible circumventing approaches are discussed. A summary of the study and implications thereof conclude this report in the end.

## Experimental Methods

### Preparation of Precursors

The details of preparing the precursors (PbI_2_ films and solutions of MABr and MABr:MAI) are given Section SI-1. Briefly, the PbI_2_ precursor solution (1.2 M) was prepared by dissolving PbI_2_ in a mixture of dimethylformamide and dimethyl sulfoxide (10:0.9 in volume ratio) and spin-coated over a cleaned fused-silica substrate at room temperature, followed by thermal annealing at 70 °C for 10 min. MABr was dissolved in isopropanol (IPA) to yield an 18 mM solution, while the MABr:MAI solutions with a series of bromide fractions were prepared similarly with a combined concentration of 18 mM. All the precursors were prepared and stored in a nitrogen-purged glovebox before perovskite growth.

### Growth of Perovskite Films

To accurately capture the evolution of the two-step solution growth of perovskites, we designed a specialized growth chamber capable of precise controlling the reaction temperature *T*_R_ and duration *t*_R_, as shown in Figure S1. Detailed fabrication instructions are provided in Section SI-1. In the growth scenario of PbI_2_ reacting with MABr solution, the solution was preheated in the chamber to a preset *T*_R_. The PbI_2_ substrate was immersed into the solution to initiate the reaction and then lifted above the solution surface to stop the reaction, yielding a grown sample with a reaction time, *t*_R_. The perovskite growth repeated at least two times at each *T*_R_ which was varied from 30 to 60 °C. The same procedure was followed in the growth scenario of PbI_2_ reacting with MABr:MAI solution, except the growth was proceeded at *T*_R_ = 30 °C only. After the reaction was stopped, the grown sample was immediately rinsed in pure IPA shortly and then spun with a spin-coater to remove residual MABr or MABr:MAI solution. No postannealing was performed to preserve the sample’s original state. All the samples were then stored at room temperature in the glovebox before characterization.

### Characterizations and Analyses

The PbI_2_-coated substrate and the grown perovskite sample were examined by XRD and UV–vis spectrophotometry. XRD was performed with an X-ray diffractometer (D8 Endeavor, Bruker) with Cu Kα radiation of λ_XRD_ = 1.54056 Å, a rotational step size of 0.01°, and a signal integration time of 0.1 s. The angular peak positions 2θ of XRD were calibrated for the zero shift using the relationship between peaks with Bragg’s law and calculated the lattice constant *a* (detailed in Section SI-2). UV–vis spectrophotometry was carried out with a commercial spectrophotometer (V-670, JASCO) in conjunction with a 150 mm integrating sphere (ILN-725, JASCO) to determine the pure absorbance of the sample that may contain a rough surface. The bandgap energy (*E*_g_) of the sample was obtained from the onset of the sample absorbance with respective to photon energy *E*, *A*(*E*), with a procedure described Section SI-3 because the commonly adopted Tauc-plot method involves several limitations that are associated with bandgap nature (direct vs indirect), sample crystallinity, and homogeneity and thus often produces irreproducible results.^48^ Although *E*_g_ was consistently extracted from the samples prepared in the same condition, the empirical onset method may cause some deviation in the extracted *E*_g_ value.

The detailed characterization results of the PbI_2_-coated fused-silica substrate were described in Section SI-4. In sum, its steep absorption edge near 515 nm corresponds to a bandgap energy of 2.4 eV.^49,50^ The XRD profile exhibits an XRD peak at 2θ ∼ 12.67° corresponding to the (001) lattice planes of PbI_2_. The film thickness measured with a stylus profiler (Dektak 6M, Bruker) is 200 ± 20 nm. The extracted absorption coefficient at 500 nm is around 1.1 × 10^5^ cm^–1^, which is slightly lower than that obtained from single-crystal PbI_2_ (1.38 × 10^5^ cm^–1^).^49,50^ The difference reflects the mesoporous structure of the grown PbI_2_ film^41,51^ that is supported by the characterization results of scanning electron microscopy and atomic force microscopy, shown in Figure S3c and d.

The bromide composition, *x*, of each grown MAPbBr*_x_*I_3–*x*_ sample was extracted independently from its XRD profile and absorption spectrum with the use of the relationships of
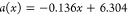
1and
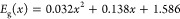
2respectively.^52^ The *x* values extracted from XRD and UV–vis spectrophotometry were compared in Figure S4a and differ by <0.2. Since the former method reveals only the crystalline portion of the sample while the data obtained with the latter one may include the amorphous portion, the comparison can verify the evolution of the halide composition during the growth process with the two independent characterization methods but also can monitor the emergence of any amorphous constituent. Finally, the extracted *x* values from three independently prepared samples showed a deviation of ∼0.12, exemplified in Figure S4b, indicating the effective control of the sample preparation procedure.

## Results and Discussion

The reaction progression in the reaction scenario of PbI_2_ reacting with MABr solution (abbreviated as PbI_2_–MABr) are prototyped by the XRD profiles and UV–vis absorption spectra of the samples grown at 30 °C at different reaction times, *t*_R_s. A three-stage model is proposed to interpret the experimental findings at this reaction temperature, *T*_R_. Its generalization is then examined with the experimental results obtained at other *T*_R_s. The rate constants of depletion of PbI_2_, growth of perovskite and halide exchange thus determined at all the *T*_R_s are used to extract their corresponding activation energies. The three-stage model is then generalized to the reaction scenario of PbI_2_ reacting with the MABr:MAI solution (abbreviated as PbI_2_–MABr:MAI). The fabrication guidelines of MAPbBr*_x_*I_3–*x*_, for *x* varying from 0.1 to 2.9, developed based on the films grown with the two reaction scenarios, are presented. Lastly, the issues involved in this study are discussed.

### Scenario I: PbI_2_–MABr

**Figure 1:**
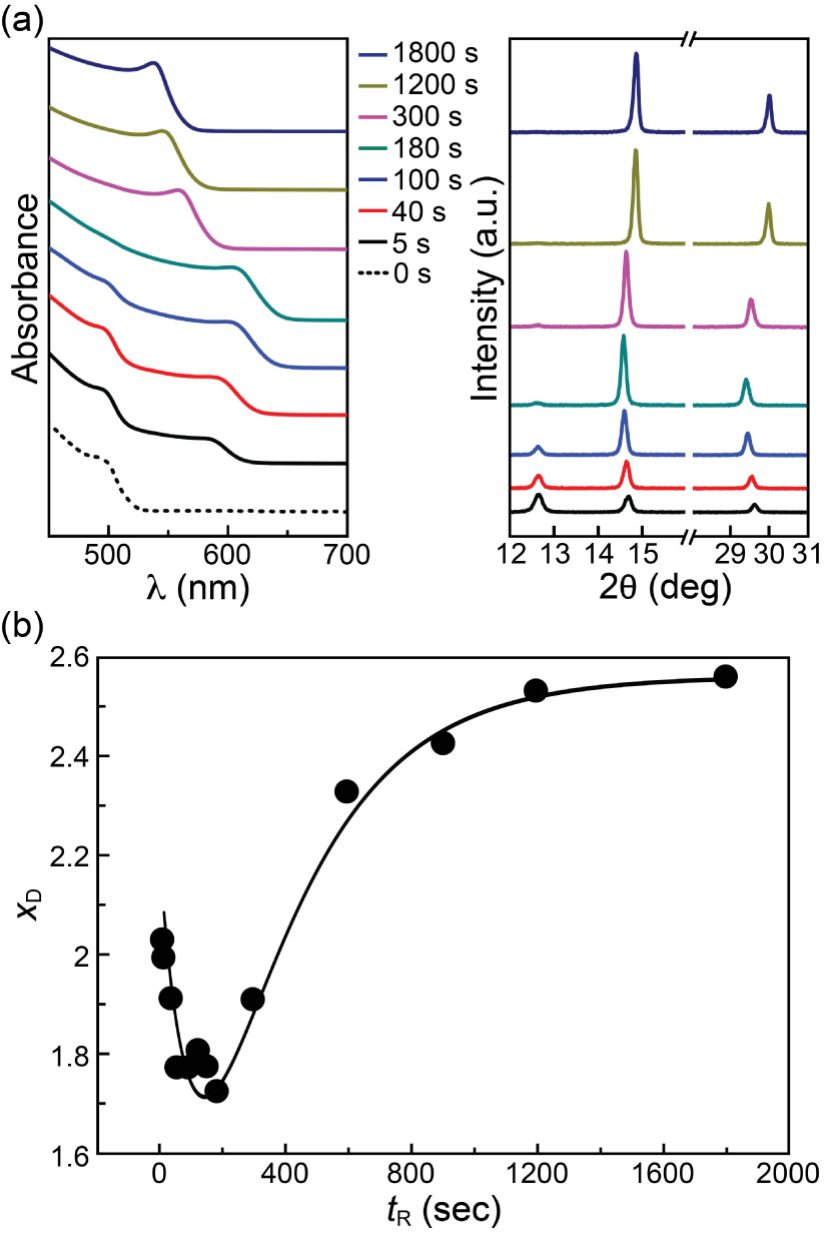
(a) UV–vis absorption spectra and X-ray diffraction profiles of grown samples at different reaction times (*t*_R_s) at reaction temperature *T*_R_ = 30 °C; (b) Br composition in MAPbBr*_x_*I_3–*x*_ extracted from XRD profiles, *x*_D_, as a function of *t*_R_. The solid line in (b) is a guide to the eyes.

At a reaction temperature (*T*_R_), the sample grown with each reaction time (*t*_R_) was characterized with X-ray diffraction (XRD) to obtain its XRD profile, *I*_D_ (2θ), and with UV–vis spectrophotometry to obtain its absorption spectrum, *A*(λ) or *A*(*E*), where θ, λ and *E* are X-ray diffraction angle, photon wavelength, and photon energy, respectively. Figure 1 shows archetypal *A*(λ)s and *I*_D_ (2θ)s of the samples grown at different *t*_R_s with *T*_R_ = 30 °C. At *t*_R_ = 5 s, an absorption feature emerges at ∼600 nm. This long-wavelength feature is red-shifted with *t*_R_ and reaches a longest wavelength of ∼640 nm (∼1.95 eV) at *t*_R_ = 180 s. It is subsequently blue-shifted with *t*_R_ and finally reaches ∼560 nm at *t*_R_ = 1800 s. In contrast, the absorption feature of PbI_2_ at ∼520 nm decreases in its strength with *t*_R_ and diminishes at *t*_R_ ≥ 180 s. The disappearance of the 520 nm feature is coincident with the appearance of the longest-wavelength absorption feature at 640 nm. As to the XRD profiles, at *t*_R_ = 5 s, two XRD peaks emerge at 2θ = 14.69° and 29.61°, corresponding to the (100) and (200) diffraction peaks of the pseudocubic perovskite phase, respectively,^53^ while the (001) peak of PbI_2_ at 2θ = 12.67° is still visible. The 2θ of the perovskite (100) peak decreases with *t*_R_, reaches its minimal value of ∼14.6° at *t*_R_ = 180 s; it then increases with *t*_R_ and finally reaches 2θ ≈ 15° at *t*_R_ = 1800 s. In comparison, the (001) peak of PbI_2_ is decreased in its strength with *t*_R_ and diminishes at *t*_R_ ≥ 180 s, behaving similarly as the absorption feature of PbI_2_ described above. According to the results of the XRD and UV–vis spectrophotometry above, two inferences can be made readily. First, the symmetric shape of the XRD peaks and their patterns indicate that the grown films are uniform pseudocubic-phase MAPbBr*_x_* I_3–*x*_ crystals that concurrently exhibit well-behaved absorption edge near bandgap (*E*_g_) in their respective absorption spectra. Second, according to the absorption edges of MAPbBr_3_ and MAPbI_3_ at 530 and 780 nm, respectively, the initially grown perovskite at *t*_R_ = 5 s is likely MAPbBr*_x_*I_3–*x*_ with *x* > 1.5 and the Br composition then decreases with *t*_R_, attains a minimal value at *t*_R_ = 180 s, and reaches a final high value (MAPbBr_3_ alike) at *t*_R_ = 1800 s. According to the diffraction theory, the lattice constant *a* of each grown pseudocubic MAPbBr*_x_*I_3–*x*_ sample can be determined. Among the reported relations between *a* and *x*_D_ of MAPbBr*_x_*I_3–*x*_,^52,54−57^ the relation obtained by the Mosca’s group,^52^eq 1, was used to determine the dependence of *x*_D_ on *t*_R_, as shown in Figure 1b, where *x*_D_ is the bromide composition determined with XRD. Of note, *x*_D_ starts at just above 2, is then decreased quickly to 1.7 at *t*_R_ = 180 s, and finally is increased gradually toward 2.6 at *t*_R_ = 1800 s. The resultant *x*_D_ (*t*_R_) curve bears four characteristic parameters: (1) the decay time constant, τ_1_, (2) the minimal *x*, *x*_D–min_, reached at , (3) the rise time constant, τ_2_, and (4) the asymptotic bromide composition at *t*_R_ → *∞*, *x*_D–*∞*_. The Br compositions of the grown MAPbBr*_x_* I_3–*x*_ samples were also obtained with their relation with bandgaps, eq 2.^52^ The agreement is within 0.2, as exemplified in Figure S4a, indicating that the amorphous perovskite was negligible in the grown film. The behaviors shown in Figure 1 were confirmed by three independently repeated experiments, shown in Figure S4b. The *x*_D_s at different *t*_R_s were similarly extracted at other *T*_R_s. The results (Figure S5) show that both τ_1_ and τ_2_ decreases with *T*_R_, as expected, while *x*_D–min_ decreases with *T*_R_ and attains ∼1.5 at ≥40 °C. A reaction model could then be derived to explain these observations.

### Three-Stage Model

**Figure 2:**
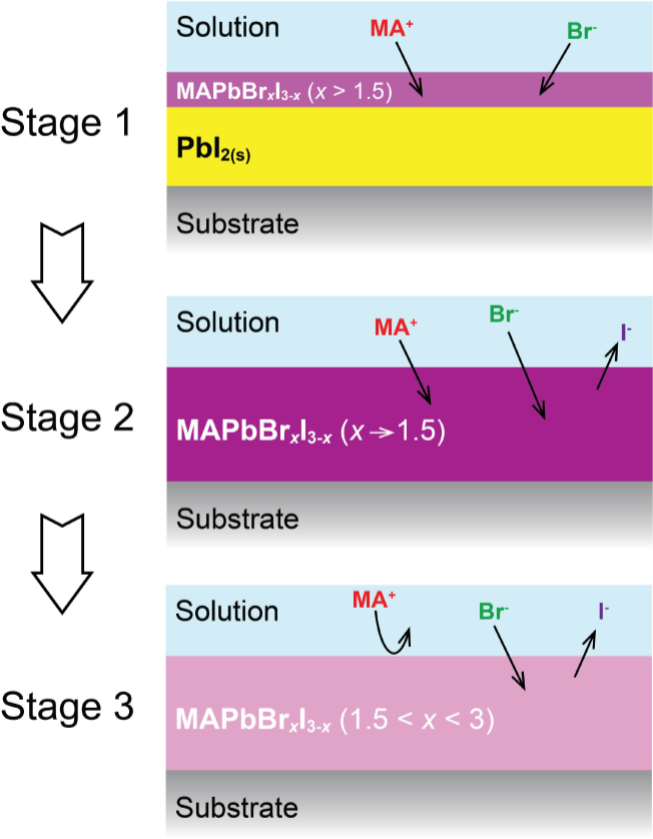
Schematic diagram of three-stage model: (1) Stage 1—a capping layer of MAPbBr*_x_*I_3–*x*_ with a high Br composition (*x* > 1.5) is formed; (2) Stage 2—PbI_2_ layer is converted to MAPbBr*_x_*I_3–*x*_ with a medium Br composition until reaching *x* ∼ 1.5; (3) Stage 3—I-to-Br exchange produces MAPbBr*_x_*I_3–*x*_ with a higher Br composition (1.5 < *x* < 3).

Based on the progression of the Br composition of the grown MAPbBr*_x_*I_3–*x*_ perovskite films presented above, the growth can be separated into three stages, illustrated in Figure 2, with the watershed reaction time, , when a minimum *x*_D_ value, *x*_D–min_, is reached. In Stage 1, a MAPbBr*_x_*I_3–*x*_ (*x* > 2.0) capping layer is promptly formed on the top surface of the PbI_2_ film within a few seconds. In Stage 2, as , *x*_D_ decreases, forming MAPbBr*_x_*I_3–*x*_ with *x* = *x*_D–min_ at . In Stage 3, as , *x*_D_ increases, forming high-*x* MAPbBr*_x_*I_3–*x*_ (*x* > *x*_D–min_), as exemplified in Figure 1b. In the initial stage, MA^+^ and Br^–^ expectantly interact with the top surface of the PbI_2_ film to destruct the PbI_2_ lattice structure and form a MAPbBr*_x_*I_3–*x*_ capping layer. Although there is no report on the dissolution of PbI_2_ in IPA, it is known that PbI_2_ tends to be dissolved in MAI solution^44^ and form iodoplumbates (e.g., PbI_3_^–^ and PbI_4_^–2^)^52^ due to the strong donor ability of I^–^.^15,58^ The recent simulation study of the formation of MAPbI_3_ by Kaiser et al.,^47^ without considering the solvent, showed that the initial nucleophilic attack by I^–^ causes structural disorder in the PbI_2_ layer, while the second nucleophilic I^–^ attack breaks two Pb–I bonds simultaneously to form [PbI_*n*_]^2–*n*^ complexes for facile nucleation of perovskite with MA^+^. If their study is conceivable, the initial appearance of MAPbBr*_x_*I_3–*x*_ with *x* > 2 in our study suggests that the stronger affinity of nucleophilic Br^–^ from MABr opens the top of PbI_2_ structure^31,59,60^ and forms high-*x* haloplumbates [PbBr*_x_*I_3–*x*_]^−^ after breaking Pb–I bond and releasing I^–^ in solution. The process can be described as eq 3:
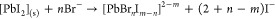
3where *m* ≥ 3 and *n* = 1, 2, .... The formed univalent haloplumbate [PbBr*_x_*I_3–*x*_]^−^ hypothetically reacts with MA^+^ to form MAPbBr*_x_*I_3–*x*_ on the surface within a few seconds:
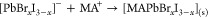
4

In Stage 2, the *x* value drops to *x*_D-min_ ∼ 1.5, i.e., the composition of Br is close to that of I in MAPbBr*_x_*I_3–*x*_ at , indicating that more I^–^ ions released from the destruction of surface PbI_2_ lattice are incorporated into the conversion reaction: The attack of the PbI_2_ lattice is more by I^–^ and less by penetrated Br^–^ to form [PbBr*_x_*I_3–*x*_]^−^, followed by the formation of MAPbBr*_x_*I_3–*x*_ with a smaller *x*. Since the initially grown Br-rich MAPbBr*_x_*I_3–*x*_ capping layer bears less porosity caused by the volume expansion of the perovskite lattice with respect to the PbI_2_ lattice^61,62^ and MA^+^ is more mobile than Br^–^ due to their mass and charge difference, the conversion reaction, eq 4, occurring at the interface between the Br-rich MAPbBr*_x_*I_3–*x*_ capping layer and the PbI_2_ lattice underneath would proceed with the deeply penetrated MA^+^ and the haloplumbate [PbBr*_x_*I_3–*x*_]^−^ formed from the destruction of PbI_2_ lattice, eq 3, gradually less with penetrated Br^–^ but increasingly more with the I^–^ released from the previously destructed PbI_2_ lattice. Besides the fact that the dense capping layer slows the diffusion of Br^–^ to the perovskite/PbI_2_ interface and the diffusion of I^–^ away from the interface into the solution, the accumulated I^–^ ions on the perovskite/PbI_2_ interface not only mitigate the penetration of Br^–^ ions but also attract MA^+^ ions along the narrowed canals in the expected compact perovskite capping layer. As a consequence, the I composition of the grown perovskite in Stage 2 increases with *t*_R_, as exemplified in Figure 1b. As the PbI_2_ lattice diminishes, the whole film is coincidently converted to a perovskite film while the Br^–^ ions, which penetrate into the grown perovskite layer through the channel between perovskite grains, would undertake an anion exchange reaction with the iodine in the grown perovskite grains, as the start of Stage 3, resulting in a high-*x* MAPbBr*_x_*I_3–*x*_ film. Ultimately, the Br composition of the grown perovskite approaches 1.5 when the PbI_2_ film is depleted, indicating that approximately 75% of iodine atoms from the PbI_2_ film participate in the construction of the perovskite lattice, while the rest of the iodine atoms become ions diffusing into the solution if they are not trapped at the boundary between perovskite grains. The halide ion exchange was investigated in several previous studies.^63,64^ In a Br/I exchange reaction of MAPbX_3_ (X = Br and I), Pellet et al. showed that the exchange process is limited by the halide diffusion within the perovskite lattice rather than ion transport in the solution or by the interfacial halide exchange at the surface.^63^ Notably, Ghasemi et al. recently examined the lateral diffusion of I^–^ along the surface of MAPbBr_3_ with secondary ion mass spectrometry and found that the diffusion along grain boundary is much faster than that in grain volume.^64^

The three-stage model derived from the PbI_2_–MABr growth is different from the model proposed by Fu et al.^25^ who also monitored the two-step growth of MAPbBr*_x_*I_3–*x*_ with PbI_2_ reacting with MABr in IPA at room temperature with XRD and observed MAPbBr*_x_*I_3–*x*_ at *t*_R_ = 1 min and MAPbBr_3_ at *t*_R_ = 4 h. They posited that this thin capping layer of MAPbBr*_x_*I_3–*x*_ with a high *x* at the initial stage would block permeation of MABr. However, they failed to observe the construction of MAPbBr*_x_*I_3–*x*_ with quickly decreasing *x* and the subsequent growth of MAPbBr*_x_*I_3–*x*_ with slowly increasing via the I-to-Br exchange reaction. Accordingly, they conjectured that the dissolution of the PbI_2_ layer and the crystallization of MAPbBr_3_—so-called the dissolution–crystallization model. That is, they missed the critical second and third stages in our model. Chauhan et al.^42^ applied the same dissolution–crystallization model to explain their in situ optical absorption results. Moreover, no detailed mechanism was revealed, and no kinetic analysis was performed in these two studies.

### Kinetic Analysis

**Figure 3:**
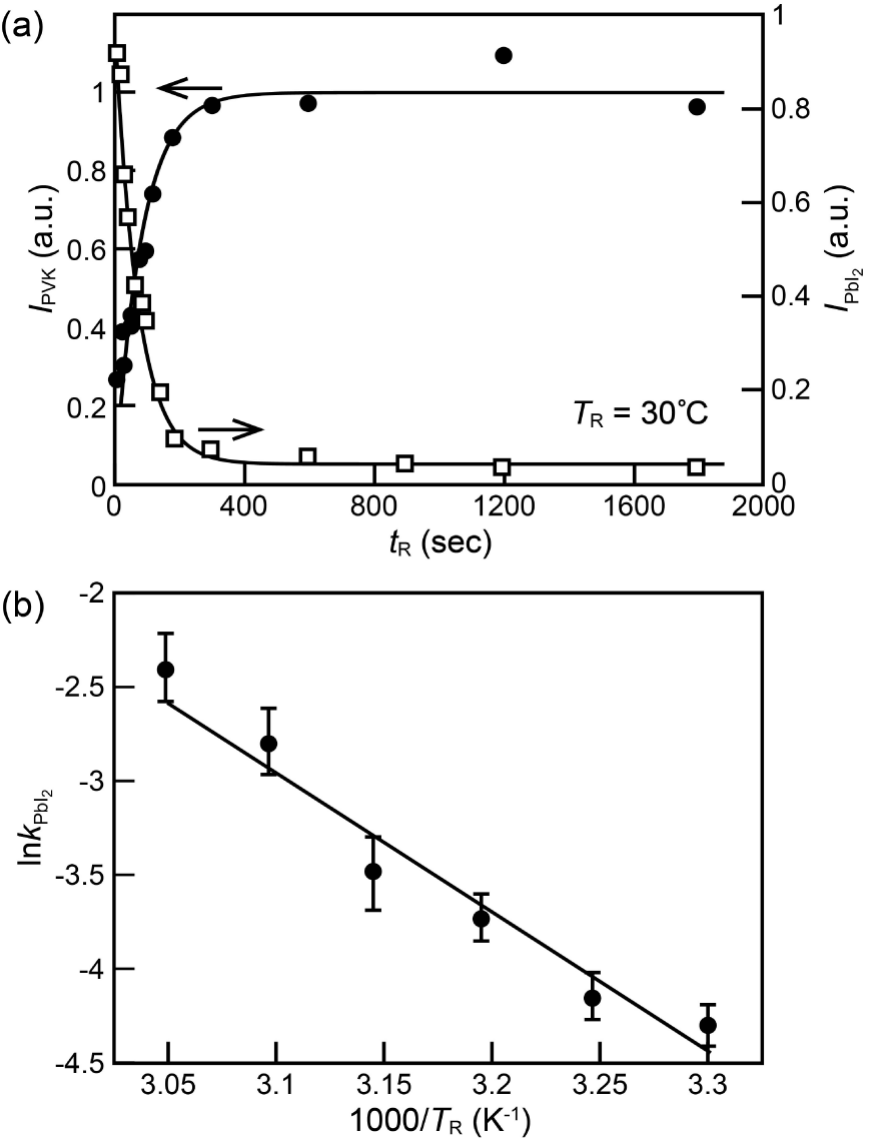
(a) Extracted areas of (001) XRD peak of residual PbI_2_, s (open squares), and those of (100) XRD peak of perovskite, *I*_PVK_s (filled circles), obtained with a reaction temperature, *T*_R_, at 30 °C plotted against reaction time, *t*_R_. The curves are the fitted curves with use of eqs 5a, 5b, respectively; (b) extracted rate constants of the depletion of PbI_2_, s, from the XRD plotted against 1000/*T*_R_ and fitted with eq 6. The line in (b) is a fitted curve to eq 6.

Based on the three-stage model, the kinetics of the perovskite formation in Stage 2 and that of the I-to-Br exchange reaction in Stage 3 can be obtained with the evolution of the XRD profile at different *T*_R_s. As an example, Figure 3a shows the dependences of the extracted peak area of the (001) XRD peak of PbI_2_, , and that of the (100) peak of MAPbBr*_x_*I_3–*x*_, *I*_PVK_, on *t*_R_ at *T*_R_ = 30 °C. Note that  decays while *I*_PVK_ coincidently rises exponentially with *t*_R_. The fitting of these two data with single exponential function, i.e., 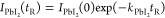
5aand
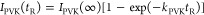
5bwhere (0) corresponds to the initial  and *I*_PVK_ (*∞*) is the final *I*_PVK_, yielded two almost identical rate constants:  = 0.013 s^–1^ and *k*_PVK_ = 0.012 s^–1^, indicating that both the depletion of PbI_2_ and the growth of perovskite behave as a direct pseudo-first-order reaction, thus amounting to the first kinetic result of this study. Therefore, no intermediate species during the reaction is needed to interpret the analysis result. All Pb atoms from the PbI_2_ template are consumed in the perovskite growth, and the supply of MA^+^ is opportune for the growth. This result is in contrast to those obtained by previous studies^41,46^ that claimed the existence of certain intermediate complex or disordered structure. We notice that our rate constant at 30 °C is significantly slower than that obtained from the two-step MAPbI_3_ growth study—MAI in IPA (38 mM) reacts with spin-coated PbI_2_ coated on mesoporous Al_2_O_3_—at 25 °C by Gratzel’s group (0.058 s^–1^).^41^ Two possible causes lead to their high growth rate. First, their higher MAI concentration, compared with our MABr concentration of 18 mM, compensates for the lower reactivity of I^–^ compared with Br^–^. Second, their PbI_2_ layer deposited on mesoporous Al_2_O_3_ may bear different characteristics (crystallinity, porosity, etc.) from ours. Unfortunately, because of the high growth rate of their studies and only three temperatures employed, no activation energy, *E*_A_, was extracted from their qualitative investigation. Our systematic temperature-dependent study (see Figure S6) on the other hand allows for extracting *E*_A_. Figure 3b shows the obtained rate constant in logarithmic scale, , plotted against 1000/*T*_R_.  shows linear relation with 1000/*T*_R_. That is, the activation energy can be obtained with the Arrhenius equation:
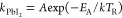
6

The fitting of the rate constant  vs 1/*T*_R_ resulted in an activation energy of 0.72 eV. This value is close to the decomposition energies of MAPbBr_3_ and MAPbI_3_ (0.62 and 0.7 eV, respectively)^65^ due to a low heat of formation from PbI_2_ with MAI to MAPbI_3_ has been reported (–0.1∼0.4 eV).^66,67^ In contrast, this activation energy is significantly smaller than the one extracted from the reaction of PbI_2_ film with MAI gas from 100 to 140 °C (*E*_A_ = 1.89 eV).^67^ The difference may come from the following three facts. First, before the reaction, MAI gas acquires thermal energy for its impingement on the PbI_2_ film to activate dissociation, while MABr dissociates readily into MA^+^ and Br^–^ in solution before the reaction.^68^ As a result, the gas-phase two-step method requires the substrate heated above 70 °C.^69−71^ Second, Br^–^ is more reactive than I^–^. Third, the solvent can expedite the penetration of the MA^+^ and Br^–^ through porous morphology of PbI_2_ and screen Coulomb repulsion between halides to facilitate the formation of haloplumbates, eq 3.

**Figure 4:**
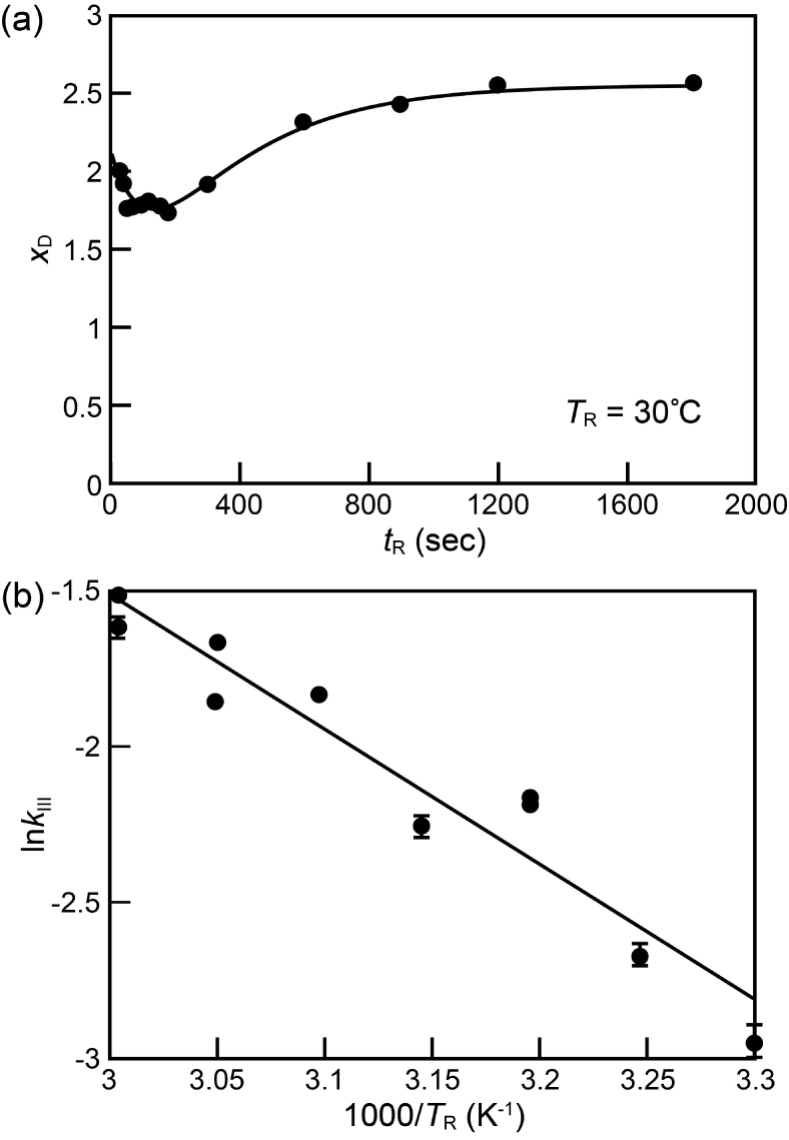
(a) Evolution of bromide composition *x*_D_ of grown perovskite at reaction temperature *T*_R_ = 30 °C fitted with eq 7; (b) extracted rate constant of anion exchange, *k*_III_, plotted against 1000/*T*_R_ and fitted with the Arrhenius equation.

The anion exchange between Br and I governs the reaction of Stage 3 in which perovskite formation is completed. Its kinetic analysis is best performed with Br composition. However, since the anion-exchange reaction starts during the formation of MAPbBr*_x_*I_3–*x*_ in Stage 2, it is imperative to retrieve the pure anion-exchange contribution from the evolution of the Br composition *x* in the grown MAPbBr*_x_*I_3–*x*_ in both Stages 2 and 3 for kinetic analysis. After zooming in on the happenings in Stages 1 and 2 of our three-stage model, once the initial high-*x* MAPbBr*_x_*I_3–*x*_ top layer is formed in Stage 1, the halide ions used for the subsequent formation of perovskite are mostly I^–^ because the Br^–^ ions in solution are essentially blocked from reaching the perovskite/PbI_2_ interface. That is, the progression of the Br composition *x* in the grown MAPbBr*_x_*I_3–*x*_ film can be considered as a combination of the initial fast decreasing *x* in Stage 2 and the subsequent slowly increasing *x* in Stages 2 and 3. As a consequence, the evolution of the Br composition, exemplified in Figure 1b, is analyzed empirically with an exponential decay and an exponential rise:
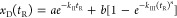
7where *k*_II_ reflects the decay of *x*_D_ due to the formation of perovskite mainly with I^–^ stemming from the dissolution of PbI_2_ in Stage 2, *k*_III_ is the apparent rate constant of I-to-Br exchange reaction in the completely grown perovskite, and *n* is an empirical number. Figure 4a exemplifies the fitting of eq 7 with the experimental data of Figure 1b. Note that the fitted curve agrees with the experimental data very well, indicating that eq 7 grasps the main kinetic characteristics of anion exchange during the formation of MAPbBr*_x_*I_3–*x*_ in Scenario I reaction. The fitting of the data with other *T*_R_s also proceeded well (Figure S5 and Table S2). The obtained *n* values in eq 7 are around 0.4, indicating that the anion exchange reaction is associated with 1D diffusion process.^46^ Last but not least, the extracted activation energy of the anion exchange reaction obtained by fitting the rate constants in Figure 4b with an Arrhenius-typed equation is 0.37 eV. This value is close to the activation energy of the anion exchange between two attached layers of MAPbI_3_ and MAPbBr_3_ (0.52 eV),^72^ those of Br ion diffusion in MAPbI_3_ through volume and grain boundary (0.61 and 0.57 eV),^64^ and that of Br and I remixing in dark between light-induced aggregated Br- and I-rich domains (0.55 eV).^73^

### Scenario II: PbI_2_–MABr:MAI

**Figure 5:**
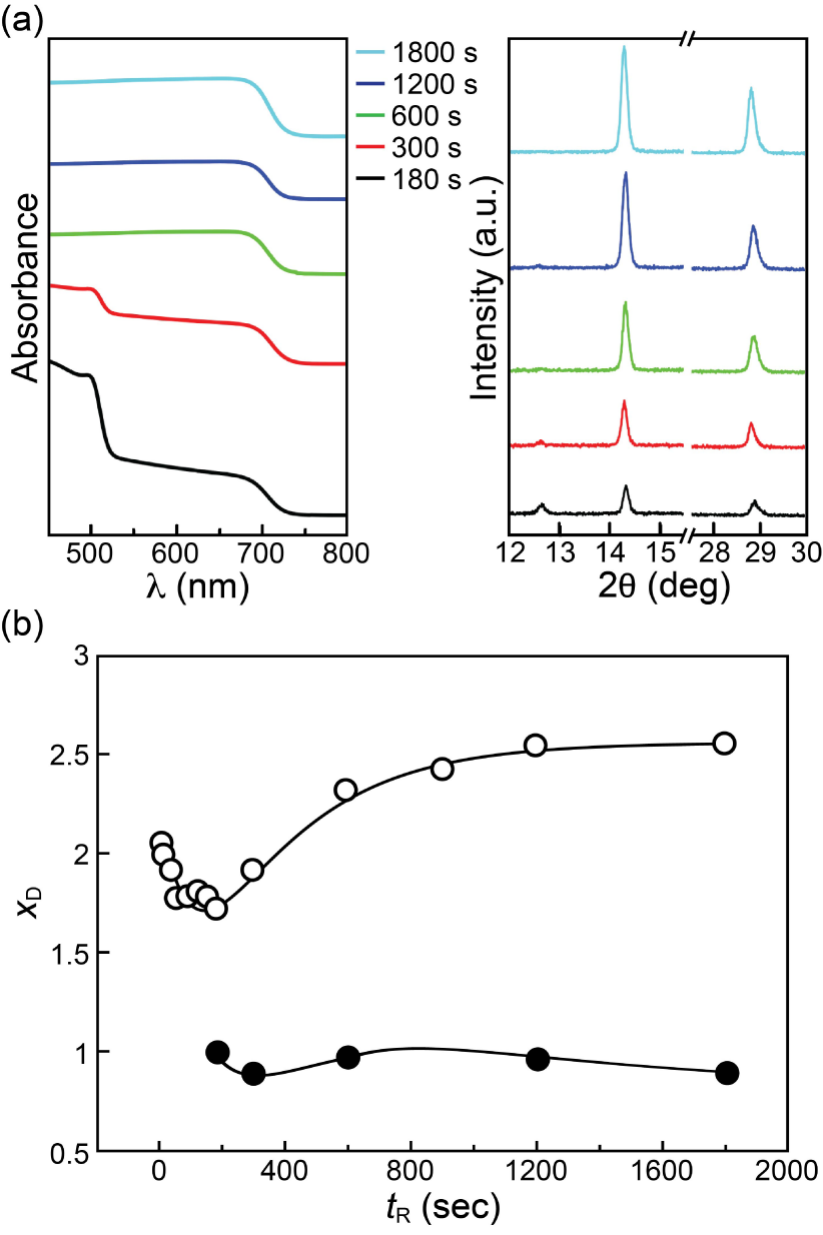
(a) UV–vis absorption spectra and X-ray diffraction (XRD) profiles of MAPbBr*_x_*I_3–*x*_ films produced with PbI_2_ reacting with a MABr:MAI solution with 50% Br^–^ fraction at different reaction times (*t*_R_s) with a reaction temperature *T*_R_ = 30 °C; (b) Br composition in MAPbBr*_x_*I_3–*x*_ extracted from XRD profiles (filled circles), *x*_D_, as a function of *t*_R_. The data in Figure 1(b) (open circles) are placed in (b) for comparison. The solid lines in (b) are guides to eyes.

Given the success of the three-stage model to interpret the experimental findings of the PbI_2_–MABr reaction scenario, how can it be extended to the other scenario of PbI_2_ reacting with a mixed solution of MABr and MAI (MABr:MAI)? The application of the three-stage model to this scenario can help to verify its effectiveness. Figure 5 shows the progression of optical absorption spectrum, *A*(λ), and XRD profile, *I*_D_(2θ), of the sample grown with a MABr:MAI solution containing 50% of Br^–^ (*f*_Br_ = 0.5) at *T*_R_ = 30 °C. At *t*_R_ = 60 s, an absorption feature emerges at ∼700 nm. This long-wavelength feature is red-shifted with *t*_R_ and reaches ∼730 nm at *t*_R_ = 300 s and is subsequently blue-shifted slightly to ∼720 nm at *t*_R_ = 600 s and remains approximately at that wavelength until *t*_R_ = 1800 s. On the other hand, the absorption feature of PbI_2_ at ∼520 nm decreases in its strength with *t*_R_ and diminishes at *t*_R_ = 600 s. Correspondingly, the (100) peak of the perovskite film in the XRD profile emerges at 2θ = 14.34° at *t*_R_ = 180 s is shifted to 2θ = 14.31° at *t*_R_ = 300 s, returns to 2θ = 14.34° at *t*_R_ = 600 s, and is gradually shifted to 2θ = 14.31° at *t*_R_ = 1800 s, while the XRD peak strength of PbI_2_ at 2θ = 12.67° decreases to nearly zero at *t*_R_ = 600 s. Figure 5b shows the extracted Br compositions of the perovskite films at these *t*_R_’s as well as those of PbI_2_ reacting with MABr for comparison. The *A*(λ) and *I*_D_ (2θ) results of the grown perovskite films with different *f*_Br_s are alike (Figure S7). The results from these two growth scenarios show similar behaviors: An initial decrease in Br composition is followed by a gradual rise. Since both Br^–^ and I^–^ are present in the precursor solution in this reaction scenario, three traits in the resultant evolution of the Br composition are expected. First, compared to the PbI_2_–MABr scenario, the initial Br composition in the PbI_2_–MABr:MAI scenario is expectantly smaller, resulting in a capping perovskite layer with narrower channels between larger perovskite grains, due to larger iodine^61,62^ and thus with less permeability for methylammonium cations and halogen anions. Second, compared with the behavior of Stage 2 in the PbI_2_–MABr scenario, the minimal Br composition of the grown perovskite films, *x*_D–min_, is smaller and the reaction time to attain it, , is longer. Third, the I-to-Br exchange in Stage 3 would proceed more slowly, and the final Br composition would be smaller. These characteristics become more significant as *f*_Br_ is smaller (Figure S8). In summary, the perovskite growth results of this scenario also effectuate the three-stage model.

### Fabrication Guidelines of MAPbBr*_x_*I_3–*x*_

**Figure 6:**
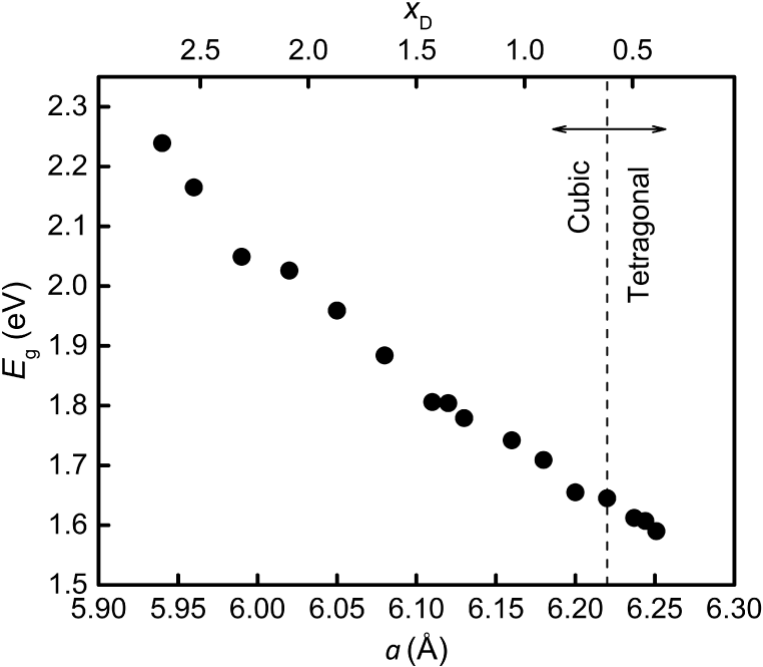
Extracted energy bandgap (*E_g_*) from UV–vis absorption spectra vs extracted lattice constant *a* from X-ray diffraction of grown MAPbBr*_x_*I_3–*x*_ with *x* ranging from 0.3 to 2.9 according to the guidelines (detailed in text). The lattice constants of the three tetragonal-phase samples were obtained by their corresponding lattice constants of the *ab* plane divided by . Equation (1)^52^ was used to obtain the relationship between *a* and *x*_D_.

All the mixed-halide perovskites grown with the two scenarios—(1) PbI_2_ reacting with MABr solution and (2) PbI_2_ reacting with MABr:MAI solution—cover the MAPbBr*_x_*I_3–*x*_ films with *x* varying from 0.1 to 2.9. Most importantly, these mixed-halide perovskite films confer sharp XRD peaks and steep absorption onsets, indicating that they are homogeneous and free of phase segregation which often occurs in the mixed-halide perovskite films produced with the one-step growth method.^12^ Our preliminary optical characterizations with photoluminescence and Raman spectroscopy also confirm the single-phase growth of mixed-halide perovskites and will be reported in future publications. Based on these results, we therefore derive two guidelines for fabricating high-quality mixed-halide perovskites. First, the PbI_2_–MABr scenario in the third anion-exchange stage produces the MAPbBr*_x_*I_3–*x*_ films covering *x* from 1.5 to 2.9, as exemplified in Figure 1b. Second, the PbI_2_–MABr:MAI scenario after the second stage produces the MAPbBr*_x_*I_3–*x*_ films with *x* varying from 0.1 to 1.5 according to the dependences of the Br composition of the MAPbBr*_x_*I_3–*x*_ film on the fraction of Br^–^ in MABr:MAI solution and on the reaction time (Figure S8). The resultant homogeneous single-phase mixed-halide perovskites, which are difficult to come by with the one-step method if not impossible, provide a great opportunity to interrogate the dependence of fundamental optoelectronic properties on halide composition, particularly the energy- and charge-transfer properties that are sensitive to material imperfections and phase segregation. Figure 6 shows the relation between the energy bandgap *E*_g_ and the lattice constant *a* of MAPbBr*_x_*I_3–*x*_ with *x* ranging from 0.3 to 2.9 that were grown according to the guidelines. The lattice constants of the three tetragonal-phase samples in the figure were obtained by their corresponding lattice constants of the *ab* plane divided by .^53^ Of note, all the grown samples are single phase and free of phase segregation according to their XRD profiles.

### Strengths and Issues

The design of this study has three strengths. First, we partially or completely replaced the halogen element in ammonium-halide solution to investigate the two-step solution-growth process of ammonium lead-halide perovskites, offering an unprecedented opportunity to decipher the dominant role of halogen from the lead-halide template in constructing the perovskite structure. Second, optical absorption spectroscopy and X-ray diffraction provide complementary characterization functions in monitoring the two-step growth process, allowing us to derive a three-stage model and quantitatively analyze the kinetics of the dissolution of the lead-halide template and the construction of perovskites. Third, the unfolded kinetic results help in developing two fabrication guidelines of single-phase mixed-halide perovskites with predictable stoichiometry. Most importantly, the mechanistic role of the lead-halide template in Stage 2 is revealed through the study design: The mesoscopic channels inside the lead-halide substrate guide the permeation of the ions in solution, while its crystallinity affects the crystal quality of the grown perovskite film.

Given the discoveries resulting from this study, there still exist several issues and limitations that are worthy of further discussion. First, in light of the revealed role of the lead-halide template in the two-step method, can the previously reported approaches to optimize its crystallinity and morphology be further improved? In our view, these efforts lacked structural and compositional characterization tools to monitor the progression of the lead halide/perovskite interface during Stage 2 of the two-step growth process, thus, mystifying the relation between the prepared lead-halide template and the grown perovskite. Commonly used X-ray diffraction bears low spatial resolution, while electron microscopy is futile at interfacial growth, leaving optical probes to be the sensible option in future studies. Second, can the exsitu characterizations of the samples performed in this study truthfully capture the optical and structural states during the reaction, given quite a few recent in situ research efforts?^74^ That is, will the samples remain their propensities after leaving away from their reaction conditions and during the characterizations? With the careful practices described in the section of Experimental Methods, the Br composition of each MAPbBr*_x_*I_3–*x*_ perovskite film determined with its bandgap and XRD profile agree within 0.2 (Figure S4), indicating that the sample remained in its state between the measuring moments of XRD and optical spectrophotometry. Furthermore, Smecca et al.^75^ showed that the XRD signal of the MAPbI_3_ film decays in air, due to decomposition to PbI_2_, with an activation energy of ∼1 eV, which is consistent with the finding by Wang et al.^76^ with spectroscopic ellipsometry, and in vacuum with an activation energy of ∼1.5 eV. Since the time for the film at 90 °C to lose 10% of its original thickness is *T*_90_ = 2000 min, the corresponding *T*_90_ at 25 °C is estimated to be >3 yr. MAPbBr_3_ is more stable, due to the suppression of MA^+^ and Br^–^ migration.^77^ That is, the degradation of our samples during the storage in the glovebox, the sample transfer, and the XRD and spectrophotometric measurements are negligible. Lastly, our samples did not exhibit any recognizable phase segregation in the XRD profiles and UV–vis optical absorption spectra, indicating no light-induced phase segregation when the samples were temporally exposed to ambient light during the measurements. Third, why was photoluminescence (PL) spectroscopy, commonly used in previous studies, not exploited to characterize the samples? PL spectroscopy is known to suffer from nonradiative recombination processes due to traps, energy transfer, etc.,^78^ thus compromising the usage of its signal for quantitative analysis. In addition, phase segregation can be induced in mixed-halide perovskites upon inappropriate photoexcitation during PL measurements,^79−81^ thus making faulty inference on the evolution of the Br composition. Fourth, although the activation energy of the dissolution of PbI_2_ lattice and the simultaneous formation of MAPbBr*_x_*I_3–*x*_ perovskite was extracted in this study, how does it depend on the preparation conditions of lead-halide substrate and on the dissolved cation and anion (e.g., MABr:MAI)? More experimental data and a comprehensive kinetic model are needed to answer these questions. Nevertheless, the derived three-stage model can provide a sensible basis to design extended experiments and to develop a more inclusive model for different reaction scenarios. Fifth, do haloplumbates or iodoplumbates exist as the intermediate species in the two-step growth process, like in the one-step growth process?^34,35^ If yes, how can they be observed? Their presence based on the optical absorption signatures observed in the one-step growth process may not necessarily reflect their participation in the growth process, because the actual crystallization process, suggested by eq 4, has never been experimentally observed. In contrast, the dissolution of the PbI_2_ lattice and the formation of the labile haloplumbates within the restricted channels in the PbI_2_ film, illustrated in eq 3, can be monitored with optical and vibrational spectroscopy during the reaction to verify the involvement of the haloplumbates, probably necessitating some special experimental design to improve their spatiotemporal resolution and to enhance their sensitivity. Of note, Yoon et al.^31^ observed absorption feature of [PbBr_3_]^−^ and [PbBr_4_]^2–^ in solution while Li et al.^33^ claimed no bromoplumbate complex was observed in a similar condition, signifying the complexity of this unsettled issue. The temporary configuration of the PbBr*_x_*I_3–*x*_···MA complex could also be captured before the perovskite lattice is formed, if it does exist. Nevertheless, the coincident processes of PbI_2_ depletion and perovskite growth observed in this study reject any delay due to such an intermediate specie. Sixth, do the top and bottom regions of the grown MAPbBr*_x_*I_3–*x*_ film show different stoichiometries according to the two fabrication guidelines described above, because of inhomogeneous I-to-Br exchange for *x* ≥ 1.5 and the different diffusivities of Br^–^ and I^–^ for *x* ≤ 1.5? Our preliminary photoluminescence (PL) study of the sample upon low-power photoexcitation at 310 nm with front- and back-surface excitation schemes showed the same PL wavelengths (Figure S9). Since the absorption depth (inverse of absorption coefficient) at this excitation wavelength for both MAPbBr_3_ and MAPbI_3_ is ∼30 nm^82^ that is significantly smaller than the thickness of the perovskite films (∼300 nm), the consistent PL wavelength obtained with these two excitation schemes indicates homogeneous stoichiometric distribution throughout the perovskite film. As a note, since the diffusion length of Br^–^ in MAPbI_3_ (>10 μm)^64^ is much longer than the film thickness of this study (∼300 nm), it is no surprise of the observed stoichiometric homogeneity. Seventh, as the MAPbBr*_x_*I_3–*x*_ perovskites could also be produced by PbBr*_y_*I_2–*y*_ substrate reacting with MAI or even MABr:MAI in solution,^83,84^ can the reaction of such scenarios be explained with the three-stage model and produce stoichiometrically uniform mixed-halide perovskites? We also monitored the perovskite growth by the PbBr*_y_*I_2–*y*_ substrate reacting with MAI solution. When the reaction proceeded to *t*_R_ = 1 min, the XRD peak of PbBr*_y_*I_2–*y*_ was found to move to the angle corresponding to the XRD peak of PbI_2_ with an amplitude greatly reduced and a small XRD peak appeared at 2θ ∼ 14.3° corresponding to pseudocubic MAPbBr*_x_*I_3–*x*_, while other new prominent peaks also emerged. Simultaneously, an absorption feature at >600 nm and another one at ∼400 nm appeared besides the feature of PbI_2_ at ∼520 nm (Figure S10). The new emergent XRD peaks, which are different from those of pseudocubic MAPbBr*_x_*I_3–*x*_ and PbI_2_, agree with the ones corresponding to crystalline PbBr_1.2_I_0.8_,^85^ while the absorption feature at ∼400 nm is consistent with its absorption spectrum.^86^ That is, the original phase of PbBr*_y_*I_2–*y*_ was transformed promptly to a mixture of the dominant PbBr_1.2_I_0.8_ phase and the PbI_2_ phase by the initial interaction between the PbBr*_y_*I_2–*y*_ substrate and I^–^. This observation is consistent with a recent study by Sheikh et al.^86^ The XRD peaks and the absorption feature of this new phase largely remained up to *t*_R_ = 10 min, while the growth of the mixed-halide perovskite was much slower than the one of PbI_2_ interacting with the same concentration of MAI solution, indicating that the dissolution of PbBr_1.2_I_0.8_ is inefficient compared with that of PbI_2_ and therefore the growth of mixed-halide perovskites is affected. Because of the involved complication in the dissolution of PbBr_1.2_I_0.8_ and PbI_2_ and the much slower growth rate, the three-stage model needs to be modified to comprehend the observations, and accordingly, this growth scenario is not recommended for the production of mixed-halide perovskites. We note that Barrit et al.^84^ recently demonstrated the production of MAPbBr_0.3_I_2.7_, MAPbBr_1.2_I_1.8_ and MAPbBr_1.8_I_1.2_ with sequential spin-coating deposition of PbBr*_y_*I_2–*y*_ films and MABr:MAI solutions, but no growth mechanism was derived to provide fabrication guidelines. Furthermore, their grazing-incidence wide-angle X-ray scattering (GIWAXS) distribution and XRD patterns exhibited some asymmetric and irregular shapes, implicating possible inhomogeneous stoichiometry.

## Conclusions

The respective roles of I in PbI_2_ substrate and Br^–^ in MABr solution in the two-step solution-growth process of MAPbBr*_x_*I_3–*x*_ were interrogated in this study. The growth evolution was monitored with ex-situ optical and structural characterizations. A three-stage model was derived based on the optical and structural characterization results to describe the growth process: (1) growth of perovskite top capping layer with high Br composition, (2) depletion of residual PbI_2_ and simultaneous growth of underneath perovskite with medium Br composition, and (3) I-to-Br exchange in the grown mixed-halide perovskite. This revelation is not possible with the growth of MAPbI_3_ or MAPbBr_3_ because there is no way to differentiate the halide X roles from those of the PbX_2_ substrate and the MAX solution. This model was equally applicable to the growth scenario of PbI_2_ reacting with MABr:MAI in solution. The results obtained allow us to propose two fabrication guidelines of MAPbBr*_x_*I_3–*x*_: (1) for *x* ≥ 1.5, the perovskite can be grown by PbI_2_ reacting with MABr in the third stage at an appropriate reaction time; (2) for *x* ≤ 1.5, the perovskite can be grown by PbI_2_ reacting with MABr:MAI with an appropriate mixing ratio and reaction time. According to the presented optical and structural characterization results, the grown perovskites are of high crystallinity, a predictable stoichiometric ratio, and importantly superior phase homogeneity. The demystified mechanism of the two-step solution-growth method and the resultant fabrication guidelines of mixed-halide perovskites would facilitate the high-quality large-scale production of optoelectronic perovskites.

## Supporting Information Available

Experimental details (including materials, fabrication methods, characterization, and data analysis), progression of XRD profiles and UV absorption spectra of MAPbBr*_x_*I_3–*x*_ and extracted Br composition, kinetics analysis of depletion of PbI_2_ and evolution of Br composition, stoichiometric homogeneity of MAPbBr*_x_*I_3–*x*_ film revealed by photoluminescence (PDF)
