# Supplementary material for: Unraveling Halogen Role in Two-Step Solution Growth of Organic–Inorganic Hybrid Mixed-Halide Perovskites: Guidelines of Fabricating Single-Phase Perovskites with Predictable Stoichiometry
Source: ACS Omega. 2024 Jun 5;9(24):26439–49. doi: 10.1021/acsomega.4c02650 (PMC11190909; doi:10.1021/acsomega.4c02650)
Supplement: Supplementary file 1 — ao4c02650_si_001.pdf [file ao4c02650_si_001.pdf]

Supporting information

Unraveling Halogen Role in Two-Step Solution Growth of Organic-inorganic Hybrid Mixed-Halide Perovskites: Guidelines of Fabricating Single-Phase Perovskites with Predictable Stoichiometry

Ya-Rong Lee[1], Yun-Ting Chung[2], Tsung-Yu Chiang[1], Ta-Li Hsieh[1], Yi-Hang Su[1], and Juen-Kai Wang[1,3*]

[1]Institute of Atomic and Molecular Sciences, Academia Sinica, Taipei, Taiwan

[2]Department of Physics, National Taiwan University, Taipei, Taiwan

[3]Center for Condensed Matter Sciences, National Taiwan University, Taipei, Taiwan

*Corresponding author: jkwang@ntu.edu.tw

SI-1 Materials and Methods

Materials. N, N-dimethylformamide (DMF, anhydrous, 99.8%), dimethyl sulfoxide (DMSO, anhydrous, ≥ 99.9%), 2-isopropanol (IPA, anhydrous, 99.5%) were purchased from Sigma-Aldrich, while methylammonium bromide (MABr, ≥ 99.5%) and methylammonium iodide (MAI, ≥ 99.5%) were obtained from Ossila and TCI. They were used without further purification. Lead bromide ($PbBr_2$) (> 99.999%, metals basis) were purchased from Alfa Aeser. High-quality polished fused-silica substrates of a size of 20 mm × 20 mm × 0.5 mm were acquired from Unimin. The surface roughness characterized with atomic force microscopy is ~2 nm.

Preparation of PbI_2 film. Fused-silica substrates were undertaken ultrasonic cleaning sequentially in Aquet detergent, acetone and 2-propanol, followed by plasma treatment with 300±10 mTorr of oxygen (99.5%) and 40±1 Watt in a plasma cleaner (PCD150, Allreal) for 5 min. The PbI_2 precursor solution (1.2 M) was prepared by dissolving 600 mg PbI_2 in DMF/DMSO mixture solvents (10:0.9 in volume ratio) at 70°C and stirring at 500 rpm. The solution was cooled down to room temperature before use. Then 70 μl of the PbI_2 precursor was spin-coated over the cleaned substrate with a spin speed of 4000 rpm for 30 sec. The precursor film was annealed at 70°C for 10 min with a hotplate. The whole preparation procedure was performed in a nitrogen-purged glove box.

Preparation of MABr and MABr:MAI solution. The MABr precursor solution was prepared by dissolving 40 mg of MABr in 20 ml of IPA, yielding a concentration of 18 mM. The mixed MAI/MABr precursor solutions were prepared with the different mole fraction of MABr, f_{Br}, to obtain a precursor concentration of 18 mM. The precursor solutions were stirred at 500 rpm and

heated at 50°C overnight and then cooled down to room temperature before use. The whole preparation procedure was performed in a nitrogen-purged glove box.

Fabrication of perovskite. The reaction chamber designed to carry out this study is detailed in its 3D drawing (Figure S1). It is composed of chamber cap and bottom. The bottom holds a dish to contain the precursor solution. The chamber with a cap and a bottom together enclosed all the precursors to maintain the MABr concentration with an equilibrated gas-liquid phase of IPA during the growth. The petri dish with the precursor solution was held by the chamber bottom that was heated by a hotplate underneath to provide a reaction temperature T_R above room temperature but below the boiling point of IPA (82.3°C). T_R, measured with a K-type thermocouple inserted into the chamber, was found to be different from the set temperature of the hotplate by less than 1°C. A sample holder, fixed at the bottom end of a sample post, supports the placement of the sample which is realized by sliding the sample through the edge groves of the holder. The sample post, extended through a hole on the cap, is used to move the sample in and out of the precursor solution in the dish. The reaction time was precisely controlled by pushing the sample post to immerse the sample holder in the precursor solution and by pulling it up to move the sample holder out of the solution. Mixed lead halide perovskite $MAPbI_{3-x}Br_x$ films were fabricated using two reaction scenarios of the two-step solution-growth method. The chamber cap and bottom are made of aluminum, while the sample post and holder are all made of polyetheretherketone (PEEK). All the fabrication processes were also conducted in a nitrogen -purged glove box (H_2O < 0.1 ppm, O_2 < 10.0 ppm).

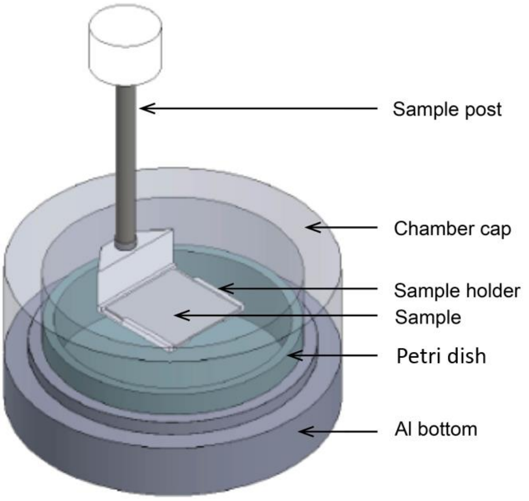

Figure S1 3D diagram of reaction chamber.

SI-2 Angular Calibration of XRD

The XRD angle θ was calibrated by using the relationship between Bragg diffraction angle and its corresponding lattice constant a. Owing to the fact that the initial angular offset $\Delta\theta$ of each sample in the sample stage is variable, the actual angle 2θ of XRD was calibrated with the relationship between the observed first- and second-order diffraction peaks, θ_1^* and θ_2^*, respectively:

$$2a\sin(\theta_1^* + \Delta\theta) = \lambda_{\mathrm{XRD}} \tag{1a}$$

and

$$2a\sin(\theta_2^* + \Delta\theta) = 2\lambda_{\mathrm{XRD}}, \tag{1b}$$

where λ_{XRD} is the X-ray wavelength. With the above two equations, the lattice constant a along a specific crystal direction and the angular offset $\Delta\theta$ were solved with the θ_1^* and θ_2^* extracted by Gaussian-fitting the first- and second-order diffraction peaks of the measured XRD profile. That is, for each XRD measurement, the procedure above was carried out to determine the actual XRD angle θ $(= \theta^* + \Delta\theta)$ and the lattice constant of the sample.

SI-3 Extraction of Energy Bandgap

In this study, the bandgap energy E_g was extracted from the absorption edge of the UV-vis absorption spectrum $A(E)$, where E is photon energy, as depicted in Figure S2. E_g is taken as the intersect between the tangent line through the inflection point of the absorption edge part and the baseline.

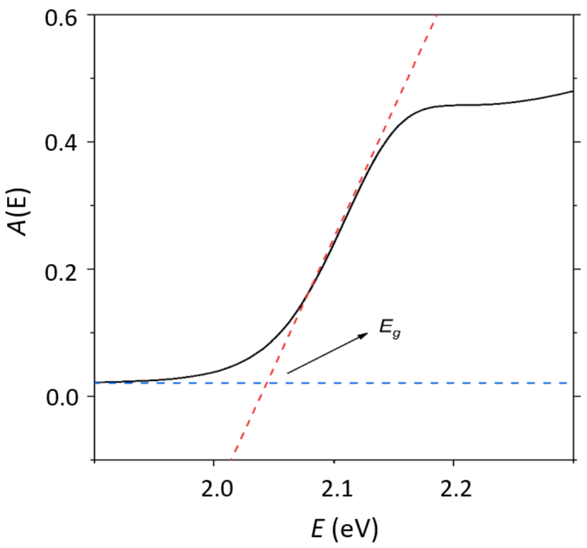

Figure S2 Absorption edge part of a typical absorption spectrum, $A(E)$, where E is photon energy. The dashed red line is the tangent line through the inflection point of the absorption edge, while the dashed blue line is the baseline.

SI-4 Characterization of PbI_2 Films

The absorption spectra of ten PbI_2 films shown in Figure S3(a) exhibit a steep absorption edge near 515 nm, corresponding to a band gap energy of 2.4 eV of PbI_2 that is consistent with previous results (e.g., Ref. 1). The variation of the absorbance above the bandgap is within 2%. The XRD profiles of seven PbI_2 films shown in Figure S3(b) exhibits an intense XRD peak positioned at $2\theta \sim 12.67°$ accompanied by less intense signals at ~25.9° and ~38.6°, originating from the (001) family of the lattice planes of PbI_2.[2] The peak at ~22.6° was originated from the XRD sample holder. The film thickness measured with a stylus profiler is 200±20 nm over four samples for scanning nine sites per sample. The larger thickness variation than the absorbance variation (2%) may be due to the measurement error of the stylus profiler. Based on the absorption absorbance and the film thickness, the absorption coefficient at 500 nm was estimated to be around 1.1×10^5 cm^{-1}, which is slightly lower than that obtained from single-crystal PbI_2 (1.38×10^5 cm^{-1}).[2,3] The difference may be partly due to the mesoporous structure of the PbI_2 film. The surface morphology of the PbI_2 film was revealed by scanning electron microscopy, as shown in Figure S3(c), presenting a mesoporous structure[4] with an average pore size of ~100 nm. Such porosity is also reflected in the measured image of atomic force microscopy, Figure S3(d), exhibiting a root-mean-square roughness of ~7 nm.

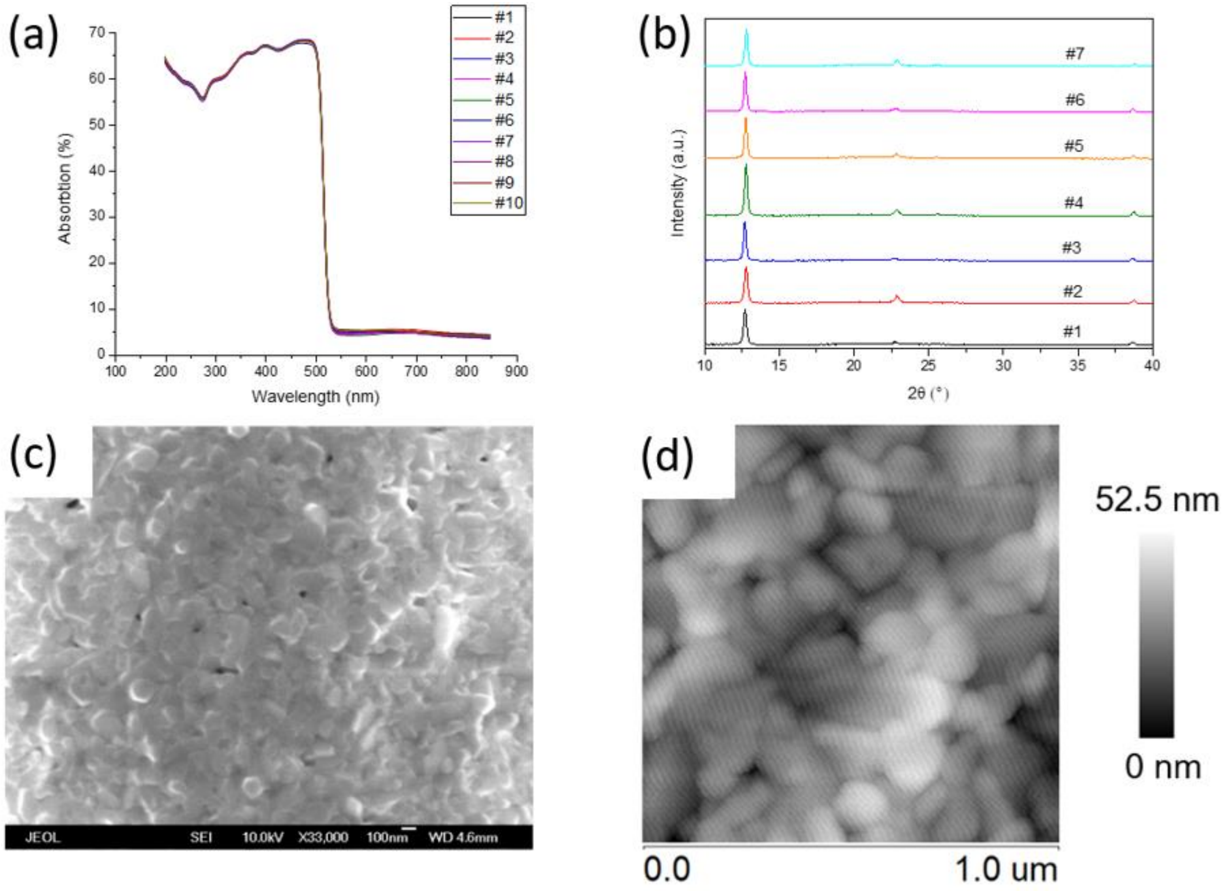

Figure S3 Characteristics of PbI_2 films: (a) UV-vis absorption spectra of ten samples, (b) XRD profiles of seven samples, (c) typical image of scanning electron microscopy, and (d) typical scanned image of atomic force microscopy.

SI-5 Extraction of Br composition

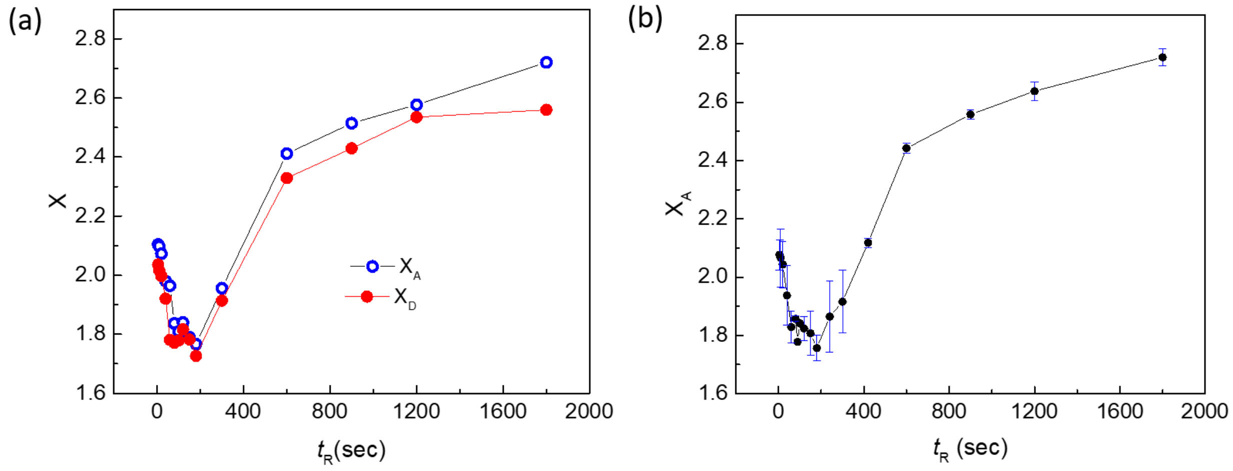

Figure S4 (a) Evolution of Br compositions determined with bandgap (x_A, blue open circles) and lattice constant (x_D, red filled circles); (b) reproducibility of x_A of three independently prepared samples.

SI-6 Evolution of Br composition of $MAPbBr_xI_{3-x}$ grown with PbI_2–MABr Scenario

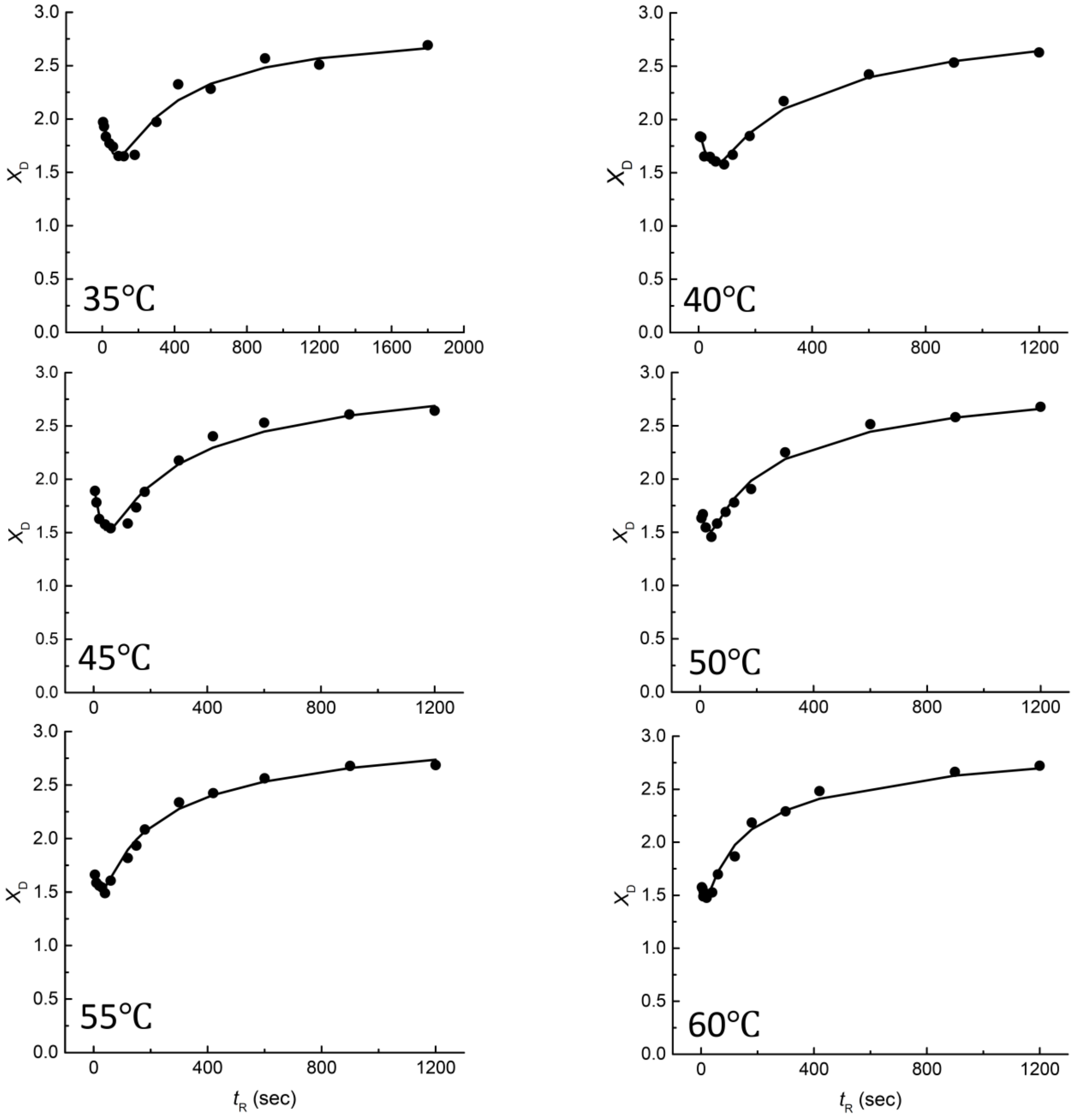

Figure S5 Br composition x_D of grown $MAPbBr_xI_{3-x}$ films extracted from their XRD profiles vs reaction time t_R at different reaction temperatures. Black curves represent the fitted curves with use of Eq. (7).

SI-7 Evolution of PbI_2

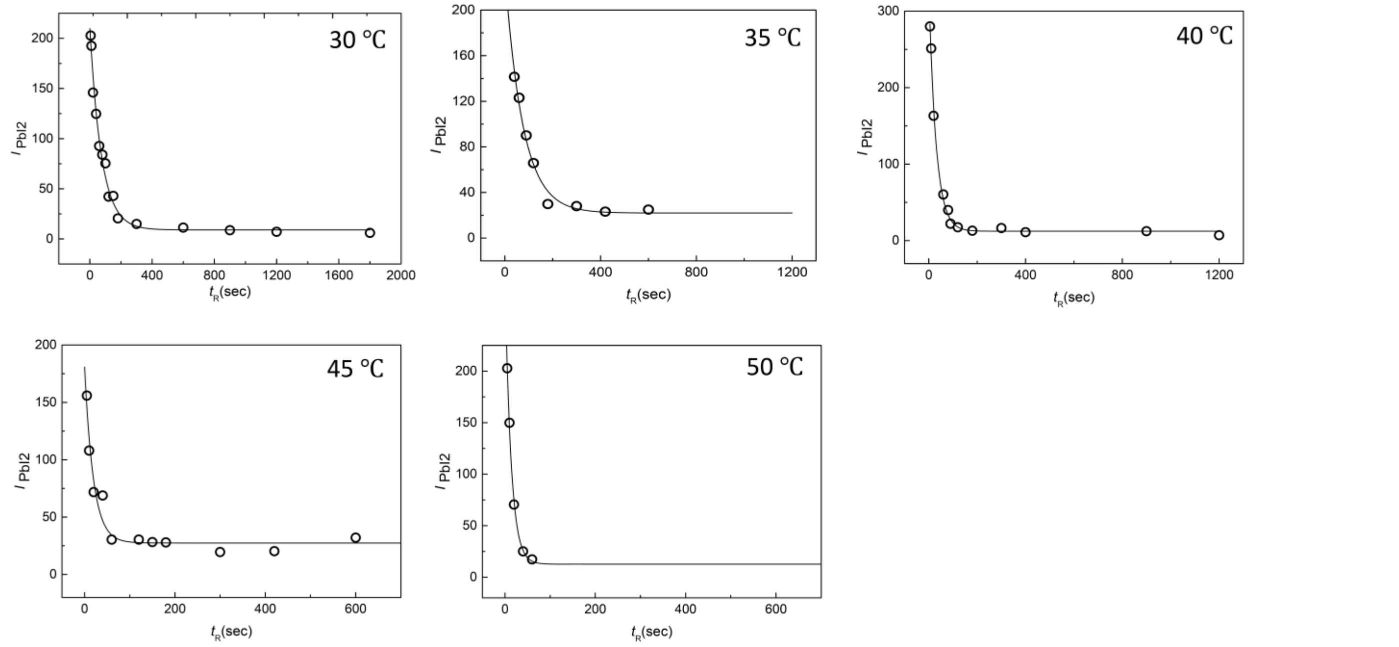

Figure S6 Area of (001) XRD peak of PbI_2, I_{PbI_2}, vs reaction time, t_R, at different reaction temperatures. The lines are the fitted curves with $I_{PbI_2}(t_R) = A \times \exp(-k_{PbI_2} t_R) + B$.

Table S1 Extracted rate constants of PbI_2 depletion based on XRD profiles, k_{PbI_2}'s, at different reaction temperatures, T_R's.

T_R (°C)	30	35	40	45	50
k_{PbI_2} (sec^{-1})	0.013 ± 0.001	0.012 ± 0.002	0.034 ± 0.002	0.052 ± 0.012	0.075 ± 0.005

Table S2 Fitted results of Br composition (x_{D}) vs. reaction time (t_{R}) with use of Eq. (7) in the main text.

T_{R} (°C)	k_{II} (sec^{-1})	k_{III} (sec^{-1})	n
30	0.016 ± 0.001	0.0058 ± 0.0006	0.57
35	0.022 ± 0.002	0.0056 ± 0.002	0.49
40	0.030 ± 0.003	0.0068 ± 0.002	0.48
40	0.034 ± 0.003	0.0053 ± 0.0002	0.41
45	0.0040 ± 0.005	0.0060 ± 0.003	0.44
50	0.057 ± 0.008	0.0071 ± 0.0003	0.37
55	0.073 ± 0.012	0.0084 ± 0.0005	0.35
55	0.061 ± 0.006	0.0083 ± 0.0004	0.39
60	0.102 ± 0.014	0.0107 ± 0.0008	0.33
60	0.091 ± 0.017	0.0092 ± 0.0009	0.34

SI-8 Evolution of $MAPbBr_xI_{3-x}$ Grown with PbI_2–MABr:MAI Scenario

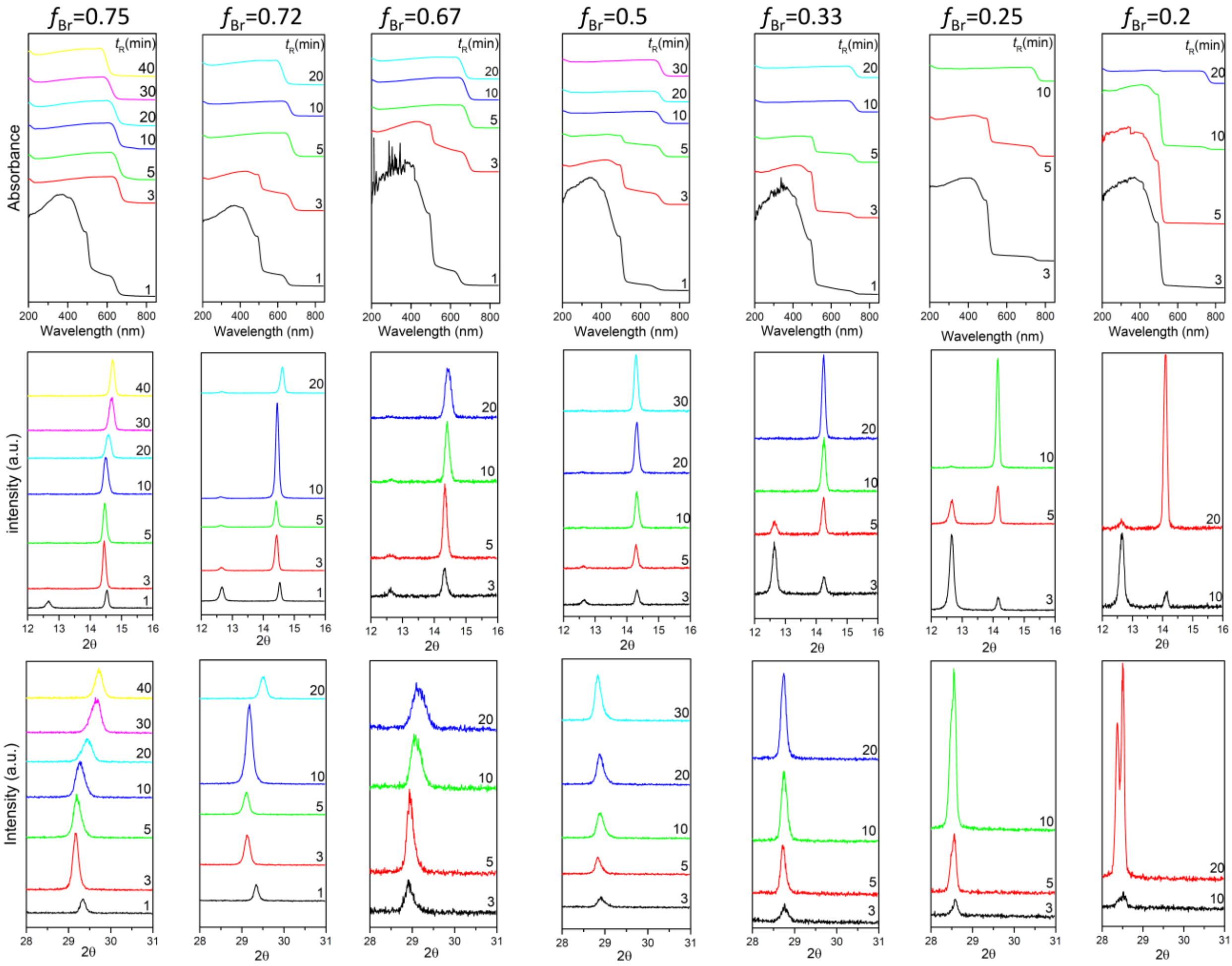

Figure S7 UV absorption spectra and XRD profiles of $MAPbBr_xI_{3-x}$ films grown by immersing PbI_2 films in different molar fractions of MABr, f_{Br}'s, in mixed solutions of MABr and MAI.

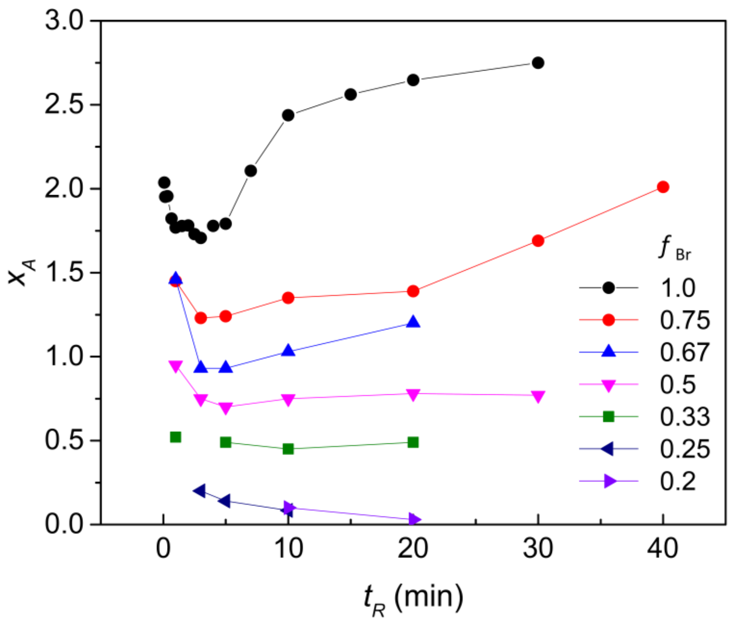

Figure S8 Evolution of Br composition x_{A}, extracted from UV-vis optical absorption spectrum $MAPbBr_xI_{3-x}$ grown with PbI_2–MABr:MAI scenario, with reaction time t_{R} for different molar fractions of MABr, f_{Br}'s.

SI-9 Front- and Back-Illuminated Photoluminescence Spectra of $MAPbBr_{1.5}I_{1.5}$

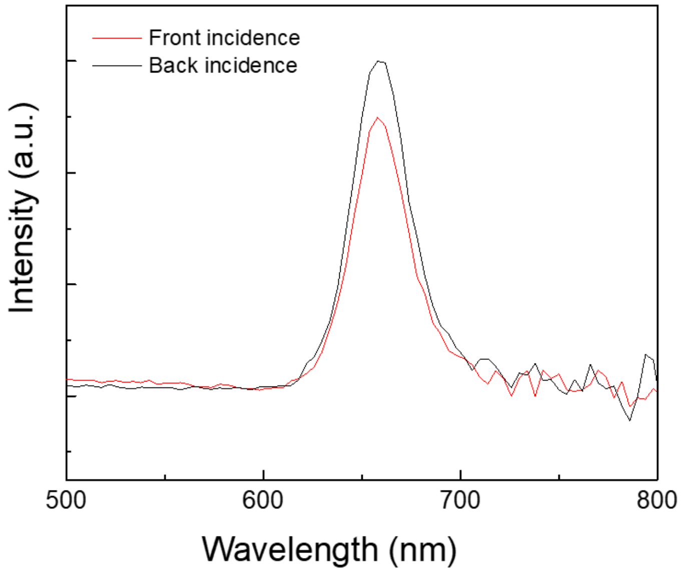

Figure S9 Photoluminescence spectra of the $MAPbBr_{1.5}I_{1.5}$ film excited at 310 nm with front- and back- illumination schemes.

SI-10 Evolution of $MAPbBr_xI_{3-x}$ Grown with $PbBr_yI_{2-y}$ Reacting with MAI

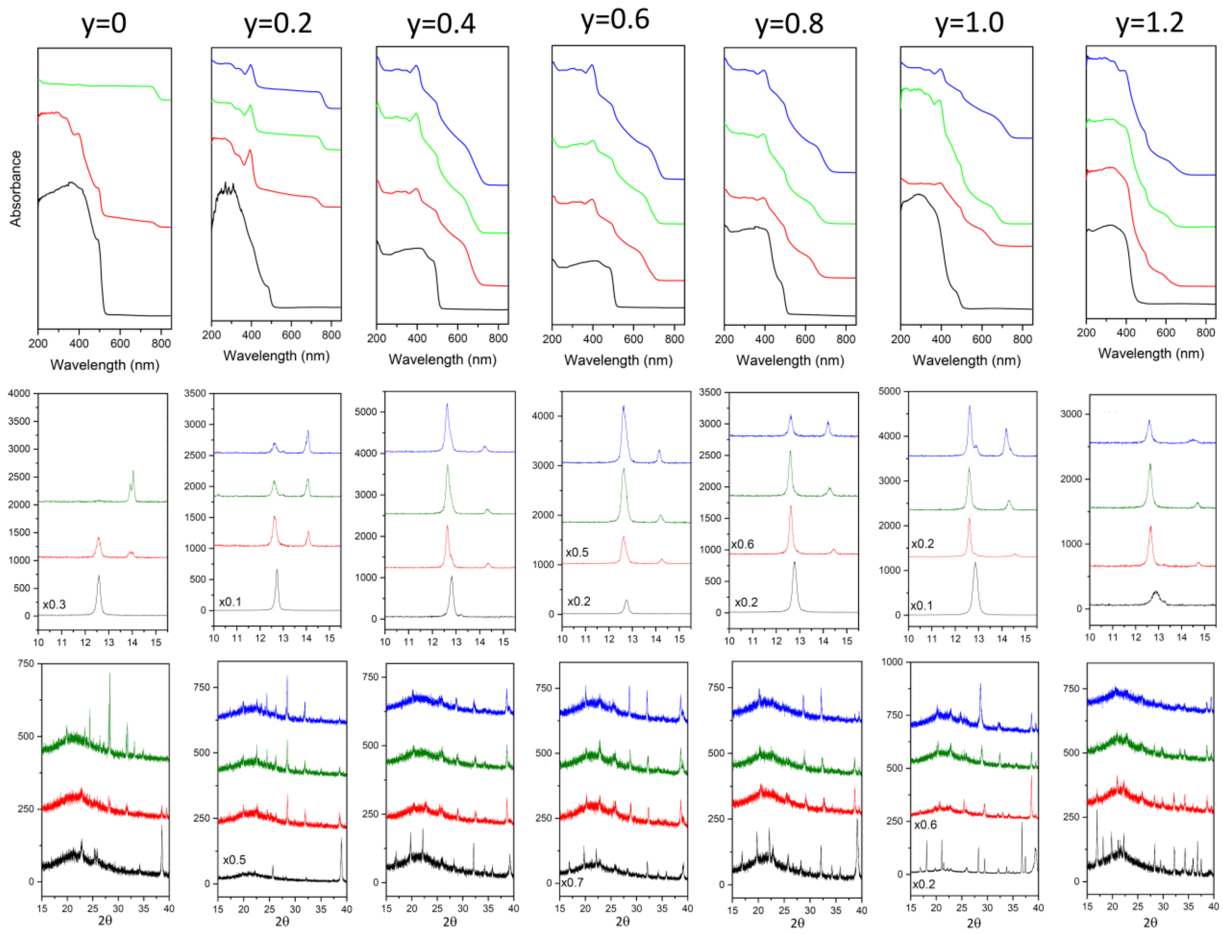

Figure S10 UV-vis optical absorption spectra and XRD profiles of $MAPbBr_xI_{3-x}$ films grown by immersing $PbBr_yI_{2-y}$ films in MAI solution (32 mM) at different reaction times, t_R's, of 0, 1, 5, and 10 min (denoted by black, red, green, and blue lines, respectively).

REFERENCES

(1) Ahuja, R.; Arwin, H.; Ferreira da Silva, A.; Persson, C.; Osorio-Guillén, J. M.; Souza de Almeida, J.; Moyses Araujo, C.; Veje, E.; Veissid, N.; An, C. Y.; Pepe, I.; Johansson, B. Electronic and optical properties of lead iodide. *J. Appl. Phys.* **2002***,* *92*, 7219–7224.

(2) Frisenda, R.; Island, J. O.; Lado, J. L.; Giovanelli, E.; Gant, P.; Nagler, P.; Bange, S.; Lupton, J. M.; Schüller, C.; Molina-Mendoza, A. J.; Aballe, L.; Foerster, M.; Korn, T.; Angel Niño, M.; de Lara, D. P.; Perez, E. M.; Fernandez-Rossier, J.; Castellanos-Gomez, A. Characterization of highly crystalline lead iodide nanosheets prepared by room-temperature solution processing. *Nanotechnology* **2017**, *28*, 455703*,*

(3) Cao, J.; Jing, X.; Yan, J.; Hu, C.; Chen, R.; Yin, J. et al. Identifying the molecular structures of intermediates for optimizing the fabrication of high-quality perovskite films. *J. Am. Chem. Soc.* **2016**, *138*, 9919–9926

(4) Lehmann, F.; Franz, A.; Többens, D. M.; Levcenco, S.; Unold, T.; Taubert, A.; Schorr, S. The phase diagram of a mixed halide (Br, I) hybrid perovskite obtained by synchrotron X-ray diffraction. *RSC Adv.* **2019**, *9*, 11151–11159.
